# Reducing diarrhoea deaths in South Africa: costs and effects of scaling up essential interventions to prevent and treat diarrhoea in under-five children

**DOI:** 10.1186/s12889-015-1689-2

**Published:** 2015-04-17

**Authors:** Lumbwe Chola, Julia Michalow, Aviva Tugendhaft, Karen Hofman

**Affiliations:** Priority Cost-Effective Lessons for Systems Strengthening South Africa (PRICELESS SA) - Medical Research Council/Wits Rural Public Health and Health Transition Research Unit (Agincourt), School of Public Health, Faculty of Health Sciences, University of the Witwatersrand, Johannesburg, South Africa

**Keywords:** Modelling, Diarrhoea, Child health, Cost analysis, South Africa

## Abstract

**Background:**

Diarrhoea is one of the leading causes of morbidity and mortality in South African children, accounting for approximately 20% of under-five deaths. Though progress has been made in scaling up multiple interventions to reduce diarrhoea in the last decade, challenges still remain. In this paper, we model the cost and impact of scaling up 13 interventions to prevent and treat childhood diarrhoea in South Africa.

**Methods:**

Modelling was done using the Lives Saved Tool (LiST). Using 2014 as the baseline, intervention coverage was increased from 2015 until 2030. Three scale up scenarios were compared: by 2030, 1) coverage of all interventions increased by ten percentage points; 2) intervention coverage increased by 20 percentage points; 3) and intervention coverage increased to 99%.

**Results:**

The model estimates 13 million diarrhoea cases at baseline. Scaling up intervention coverage averted between 3 million and 5.3 million diarrhoea cases. In 2030, diarrhoeal deaths are expected to reduce from an estimated 5,500 in 2014 to 2,800 in scenario one, 1,400 in scenario two and 100 in scenario three. The additional cost of implementing all 13 interventions will range from US$510 million (US$9 per capita) to US$960 million (US$18 per capita), of which the health system costs range between US$40 million (less than US$1 per capita) and US$170 million (US$3 per capita).

**Conclusion:**

Scaling up 13 essential interventions could have a substantial impact on reducing diarrhoeal deaths in South African children, which would contribute toward reducing child mortality in the post-MDG era. Preventive measures are key and the government should focus on improving water, sanitation and hygiene. The investments required to achieve these results seem feasible considering current health expenditure.

**Electronic supplementary material:**

The online version of this article (doi:10.1186/s12889-015-1689-2) contains supplementary material, which is available to authorized users.

## Background

Globally, one in 10 deaths in children under the age of five years results from diarrhoea, with the majority occurring in sub-Saharan Africa and South East Asia [1]. Diarrhoea is one of the leading causes of morbidity and mortality in under-five children in South Africa, however the true burden of childhood diarrhoea is not accurately known. Official data from Statistics South Africa estimate that diarrhoea accounts for approximately 20% of under-five deaths [2], but other sources estimate the burden between 8% [1] and 13% [3]. The 2010 General Household Survey (GHS), a nationally representative inquiry into the livelihood of South Africans, showed that there were over 60,000 cases of childhood diarrhoea per month and approximately 9,000 child diarrhoeal deaths in the same year [2].

Diarrhoea is closely linked to socio-economic status and has the most adverse effects in South Africa’s impoverished communities [2,4]. South African children living in poverty are approximately ten times more likely to die from diarrhoea than their more privileged counterparts [2]. Poor nutritional status, poor environmental conditions, and illnesses such as HIV/AIDS make children more susceptible to severe diarrhoea and dehydration [4]. Episodes of persistent diarrhoea also worsen a child’s condition and nutritional status due to decreased food intake and nutrient absorption [4]. In HIV-infected children, persistent diarrhoea is associated with an 11-fold increase in mortality [5]. More than 50% of South African children who died in 2012 had evidence of HIV infection or exposure, while 60% were undernourished [6].

UNICEF and WHO have stressed the importance of well-known interventions for reducing the global burden of childhood diarrhoea [4,7]. Interventions for diarrhoea prevention include vaccinations against rotavirus, cholera, typhoid and measles; micronutrient supplementation for zinc and vitamin A; prevention and treatment of comorbidities, such as HIV; exclusive breastfeeding promotion and support; adequate nutrition for mothers and children; and interventions for the provision of water, sanitation and hygiene (WASH). Diarrhoea should be treated with oral rehydration solution (ORS), zinc, continued feeding, antibiotics for dysentery, as well as improved care seeking behaviour and improved case management.

Progress is being made towards implementing these interventions. In 2009, South Africa became the only country in sub-Saharan Africa to include the rotavirus vaccine in routine child immunizations. The vaccine, which has been shown to be effective in preventing severe rotavirus diarrhoea [8], has achieved moderate coverage in South Africa (64%) [9]. The government revised its breastfeeding policy in 2011 to actively promote exclusive breastfeeding and phase out the distribution of free infant formula to babies born to HIV-positive mothers. South Africa has also achieved the water and sanitation targets for Millennium Development Goal (MDG) 7; over 90% of South Africans have access to a clean public water source and over 70% utilize a latrine or toilet [10]. However, despite achieving these goals, approximately six million households (46%) do not have access to piped water in their homes and 1.4 million households (11%) still lack access to sanitation services [11]. Furthermore, the sanitation services in over 3.8 million households (26%) in formal areas do not meet the required standards due to infrastructure deterioration [11]. Coverage remains low for many of the other recommended interventions, such as hand washing with soap and ORS. Although these health promotion interventions are affordable, there are significant challenges to increasing adoption.

The disparities in access to water and sanitation services, and the poor coverage of essential interventions contribute to the ongoing high prevalence of diarrhoea in the country. This analysis evaluates the potential impact of scaling up coverage of the recommended interventions on under-five diarrhoeal mortality in South Africa between 2014 and 2030. The potential number of lives that could be saved and the resources required for intervention scale up are assessed in order to aid priority setting and budgeting. The results of this analysis could aid South Africa’s plans to reduce child mortality in the post-2015 era.

## Methods

### Lives Saved Tool (LiST)

Modelling was done using the Lives Saved Tool (LiST), a module in the Spectrum software [12]. Version 5.07 was used (downloaded 28/11/2014). LiST is a deterministic mathematical model that compares the effect of various interventions on population level risk factors, as well as stillbirths and maternal, newborn and child deaths [13,14]. Included in the model are more than 60 interventions that have an impact on cause-specific mortality. An intervention can have an impact on single or multiple causes of death and risk factors. The outcome measures (risk factors and cause-specific mortality) change based on the level of coverage of the interventions included in the model. Increasing the level of coverage of one or more interventions can thus lead to a reduction in associated risk factors or cause-specific mortality. LiST therefore enables a user to assess the simultaneous impact of interventions on health outcomes. Intervention impact on mortality can be direct or indirect (through the reduction of risk factors). The direct impact of each of these interventions is modelled by multiplying its effectiveness estimate with the level of coverage, assuming all other interventions are kept constant. For example, an intervention with an effect estimate of 30% can avert 30% of the associated cause-specific deaths if coverage for that intervention is 100%.

When LiST analyses multiple interventions, each intervention is applied to the residual deaths from the previous intervention. This prevents double counting the number of lives saved. The model starts by applying the preventive interventions in succession, followed by the treatment interventions on remaining deaths. The total number of deaths prevented is therefore not attributable to specific interventions but rather the full intervention package [15].

**Figure 1:**
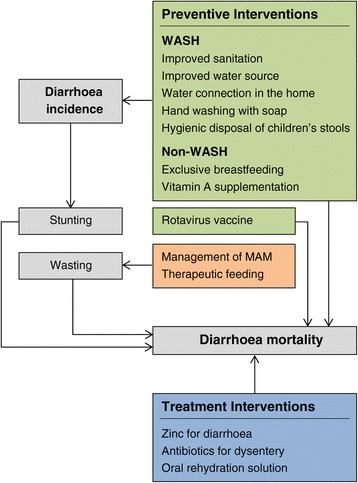
LiST interventions that impact diarrhoea mortality. Green shaded boxes = preventive interventions. Blue shaded boxes = treatment interventions. Peach and grey boxes = interventions via a risk factor pathway. WASH = interventions for water, sanitation and hygiene. MAM = moderate acute malnutrition. (Adapted from Walker C and Walker N, 2014).

LiST includes 14 interventions for the prevention and treatment of diarrhoea. Walker and Walker (2014) describe the interactions between these interventions and the modelling methods used in LiST [15]. There are 12 interventions in LiST that have a direct impact on diarrhoeal mortality. Eight of these are preventive interventions: rotavirus vaccine; vitamin A supplementation; zinc supplementation; and the water, sanitation and hygiene (WASH) programmes that include a water connection in the home or improved water source, improved sanitation hand washing with soap and hygienic disposal of children’s stools. Interventions for breastfeeding promotion, severe wasting and moderate acute malnutrition have an indirect impact on diarrhoeal mortality. The impact of breastfeeding can be modelled either as a risk factor that changes when breastfeeding promotion shifts breastfeeding rates, or as a direct risk factor for death due to the lack of appropriate breastfeeding. (In our analysis, we ramped up breastfeeding according to WHO guidelines, which recommend six months of exclusive breastfeeding and appropriate complementary feeding up to two years). LiST also includes three interventions for treating diarrhoea: zinc treatment, ORS and antibiotics for dysentery. Figure 1 (adapted to reflect the interventions addressed in our analysis) provides an overview of the intervention interactions; Zinc supplementation has been excluded from our analysis because this is not provided in South Africa.

Malnutrition is represented as a risk factor for diarrhoea mortality through the impact of stunting and wasting. Lack of appropriate breastfeeding, vitamin A supplementation and the WASH interventions influence diarrhoea incidence, which in turn affects stunting and subsequent mortality.

The effectiveness values of the diarrhoea interventions included in LiST have been reviewed by the Child Health Epidemiology Reference Group (CHERG) [16-20] and are presented in Additional file 1 [15]. The methods used in our analysis are based on similar multi-country assessments in LiST [21,22].

### Scenarios created in LiST

We assessed the impact of increasing the coverage of 13 interventions on diarrhoeal mortality. The baseline (2014) coverage levels of these interventions were reviewed and modified during a one day expert consultation hosted in South Africa. Twenty-three participants were invited from the health sector, including clinicians, academics and others in positions at national and district level. Coverage levels are indicated in Table 1. Breastfeeding prevalence at baseline was input by age group: 8% coverage of exclusive breastfeeding for infants younger than 6 months, 51% coverage of any breastfeeding for infants aged 6 – 11 months and 31% coverage of any breastfeeding for infants aged 12 – 24 months [23]. Coverage for the WASH interventions ranged from 17% for hand washing with soap to 95% for an improved water source [24]. Unchanged default LiST coverage levels have been indicated. Interventions were linearly scaled up from the baseline year 2014 until 2030, with coverage increases starting in 2015.Three scale up scenarios were implemented: in scenario one, we assumed that the coverage of all interventions increased by 10% from their baseline estimate (a fixed 0.7% increase per year); in scenario two, coverage increased by 20% (a fixed annual increase of 1.3%); and in scenario three, coverage for all interventions was increased to 99% (full coverage) in 2030. In the rest of the document, the scenarios are accordingly referred to as scenario one (10% increase), scenario two (20% increase) and scenario three (full coverage). Coverage levels for other maternal and child health interventions were not altered, in order to isolate the impact of the priority interventions for diarrhoea prevention and treatment.

**Table 1:** **Baseline and projected coverage of interventions to prevent and treat diarrhoea**

**Intervention**	**Baseline (2014)**	**Scenario 1 (+10%)**	**Scenario 2 (+20%)**	**Scenario 3 (full coverage)**
**Breastfeeding**				
Exclusive breastfeeding, < 6 months	8 [23]	18	28	99
Any breastfeeding, 6 – 11 months	51 [23]	61	71	99
Any breastfeeding, 12 – 24 months	31 [23]	41	51	99
Feeding and supplements				
Vitamin A supplementation	50	60	70	99
**WASH**				
Improved water source	95.1 [24]	99	99	99
Water connection in the home	79.2 [24]	89.2	99	99
Improved sanitation	74.4 [24]	84.4	94.4	99
Hand washing with soap	17*	27	37	99
Hygienic disposal of children's stools	40.5*	50.5	60.5	99
**Vaccines**				
Rotavirus	64 [9]	74	84	99
**Diarrhoea treatment**				
Oral rehydration solution	50	60	70	99
Antibiotics - for treatment of dysentery	80	90	99	99
Zinc - for treatment of diarrhoea	10	20	30	99
**Wasting**				
Therapeutic feeding - for severe wasting	45	55	65	99
Treatment for moderate acute malnutrition	10	20	30	99

*Default coverage level in LiST.

**Figure 2:**
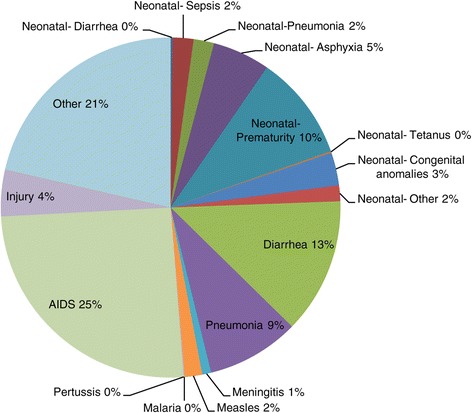
Causes of death in children under-five years, used in LiST (adapted from MRC, 2010).

The baseline mortality rates used in our analysis were 41 deaths per 1,000 live births for under-five children and 13/1,000 for neonates [3]. The causes of newborn mortality were adapted (South African Medical Research Council: Preliminary estimates for burden of disease in 2010, unpublished) estimates to fit the causal categories in LiST (Figure 2). The categories in LiST differ slightly from those presented by the MRC. For example, neonatal diarrhoea is not reported separately in the MRC BOD, but rather combined with under-five diarrhoeal deaths. Therefore, we separated these using the default proportions in LiST.

### Modelling the cost and impact of diarrhoea interventions

Modelling of costs was done using the costing module in LiST, with the most recently available data. The module uses an ingredients approach to costing, based on four components: personnel and labour; drugs and supplies; other recurrent costs; and capital costs. Staff remuneration is based on current salary structures of health workers in South Africa. Salary increases were not applied. The unit costs of drugs and supplies are based on international drug prices from UNICEF and the Management Sciences for Health International Drug Price Indicator [25,26]. The unit costs found in LiST were comparable to South African prices of drugs and supplies requested for tender by the Department of Health [27].

The unit costs for WASH programmes are not included in LiST. We estimated these costs using data available from the South African Department of Water and Sanitation [28,29]. A home water connection includes water piped into either the home or yard. This is reflected in the average cost estimate of US$480 per household (adjusted for inflation). The cost for improved sanitation was estimated using the proportion of South Africans with access to dry and wet sanitation, (60% and 40%, respectively) [10]. Wet sanitation includes various types of flush latrines and dry sanitation includes pit latrines (with and without ventilation), chemical toilets and bucket toilets. The average household cost for sanitation was approximately US$900 (adjusted for inflation).

Recurrent costs related to hospitalization and outpatient visits were not included. Recurrent costs include personnel training, gasoline, building rent, office supplies and promotional activities [30,31]. These were outside of the scope of the analysis. In addition, costs estimated in LiST exclude infrastructure development, such as building clinics [30]. All costs were adjusted to 2014 US dollars. Per capita costs use the 2014 South African population estimate of 54 million [32].

Intervention impact was measured in terms of diarrhoeal deaths averted. First, we calculated the expected number of deaths (and cases) at the current (baseline) level of intervention coverage. Second, the number of deaths (and cases) was recalculated for the three intervention scale up scenarios. Deaths averted (or additional lives saved) were then estimated by subtracting the numbers of deaths at baseline from the deaths at increased coverage (the same methodology was used to estimate the number of diarrhoea cases averted).

Ethical review board approval was not required for this analysis as no human subjects were involved.

## Results

### Cases averted in 2030

At baseline, the total number of diarrhoea cases was estimated to be 13 million. Intervention scale up would avert approximately 5.3 million in scenario three (41% decline), 4.5 million in scenario two (35% decline) and 3 million diarrhoea cases in scenario one (23% decline).

### Deaths averted in 2030

**Figure 3:**
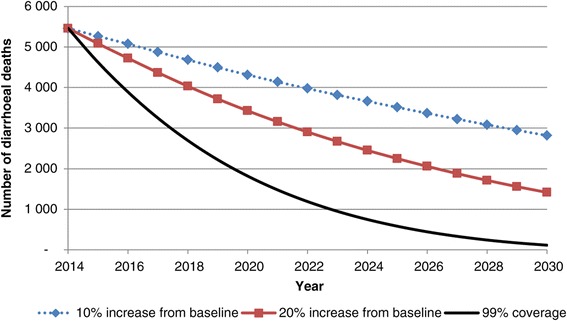
Estimated diarrhoea deaths per year (children 0–60 months) with different scenarios of increased intervention coverage.

Figure 3 illustrates the projected number of deaths due to diarrhoea in each scenario. At baseline (2014), the total number of diarrhoea deaths was estimated to be about 5,500. After scale up of diarrhoea interventions to full coverage (scenario three), the number of deaths would reduce to about 100 in 2030 (98% decline). This is compared to 1,400 deaths projected in 2030 in scenario two (74% decline) and 2,800 in scenario one (48% decline).

Table 2 shows the additional diarrhoeal deaths prevented by each intervention in the three scenarios in 2030. The percentages in the table indicate the proportion of deaths averted by each intervention for a particular scenario. For example, in scenario three, hand washing with soap averts 1 286 diarrhoeal deaths, which constitutes 25% of the deaths averted. Since water connection in the home is a subset of improved water source, the deaths averted and costs attributed to the two interventions have been combined. The total additional deaths averted were approximately 5,100 in scenario three, 3,700 in scenario two and 2,300 in scenario one.

**Table 2:** **Projected additional diarrhoeal deaths prevented (2030)**

**Interventions**	**Deaths averted**		
	**Scenario 1**	**Scenario 2**	**Scenario 3**
Hand washing with soap	243 (10%)	422 (11%)	1 286 (25%)
Breastfeeding	214 (9%)	370 (10%)	1 034 (20%)
Access to safe water*	656 (28%)	1 100 (29%)	818 (16%)
ORS - oral rehydration solution	557 (24%)	704 (19%)	518 (10%)
Hygienic disposal of children's stools	101 (4%)	176 (5%)	382 (8%)
Improved sanitation - Utilization of latrines or toilets	229 (10%)	488 (13%)	363 (7%)
Vitamin A supplementation	87 (4%)	150 (4%)	286 (6%)
Zinc - for treatment of diarrhea	78 (3%)	97 (3%)	130 (3%)
Rotavirus	59 (3%)	103 (3%)	135 (3%)
Therapeutic feeding - for severe wasting	57 (2%)	70 (2%)	70 (1%)
Treatment for moderate acute malnutrition	9 (0.4%)	12 (0.3%)	20 (0.4%)
Antibiotics - for treatment of dysentery	47 (2%)	56 (1%)	17 (0.3%)
**Total**	2 337 (100%)	3 748 (100%)	5 059 (100%)

*Access to safe water includes both an improved water source and a water connection in the home.

### Intervention costs

With intervention coverage at 99% in 2030, total intervention costs were estimated to be US$2.6 billion, representing an incremental cost of US$960 million per year (Table 3). The total costs (incremental costs) were US$2.5 billion (US$830 million) in scenario two and US$2.2 billion (US$510 million) in scenario one. This represents per capita costs of US$49 for scenario three, US$47 for scenario two and US$41 for scenario one. The incremental costs per capita were US$18 in scenario three, US$15 in scenario two and US$9 in scenario one. The WASH interventions accounted for over 90% of the total costs in all three scenarios; the two most expensive were improved sanitation and access to safe water. The total costs (incremental costs) of non-WASH interventions were approximately US$260 million (US$170 million) in scenario three, US$150 million (US$60 million) in scenario two and US$140 million (US$40 million) in scenario one. This represents per capita costs of US$4.9 in scenario three, US$2.8 in scenario two and US$2.5 in scenario one. The incremental costs per capita range from US$3 in scenario three to less than US$1 in scenarios one and two.

**Table 3:** **Projected intervention costs in 2030 (2014 US$)**

	**Scenario 1**		**Scenario 2**		**Scenario 3**	
	**Total costs**	**Incremental costs**	**Total costs**	**Incremental costs**	**Total costs**	**Incremental costs**
**WASH**						
Improved sanitation	1 304 343 520 (59.6%)	297 114 644 (58.4%)	1 530 490 650 (60.9%)	523 262 424 (62.7%)	1 531 657 562 (57.9%)	524 428 690 (54.4%)
Access to safe water*	735 212 909 (33.6%)	163 366 833 (32.1%)	816 261 686 (32.5%)	244 415 957 (29.3%)	816 884 039 (30.9%)	245 037 965 (25.4%)
Hand washing with soap	4 435 223 (0.2%)	1 988 942 (0.4%)	6 022 585 (0.2%)	3 599 383 (0.4%)	16 126 764 (0.6%)	13 703 561 (1.4%)
Hygienic disposal of children's stools	8 295 509 (0.4%)	2 467 605 (0.5%)	9 847 740 (0.4%)	4 074 817 (0.5%)	16 126 764 (0.6%)	10 353 840 (1.1%)
**Sub total**	2 052 287 161 (93.8%)	464 938 024 (91.5%)	2 362 622 661 (94%)	775 352 580 (92.9%)	2 380 795 129 (90.1%)	793 524 057 (82.3%)
**Non-WASH**						
Treatment for moderate acute malnutrition	25 051 124 (1.1%)	11 841 473 (2.3%)	37 300 440 (1.5%)	24 205 350 (2.9%)	123 459 675 (4.7%)	110 364 586 (11.4%)
Therapeutic feeding - for severe wasting	34 412 469 (1.6%)	4 719 079 (0.9%)	40 369 964 (1.6%)	10 934 337 (1.3%)	61 670 496 (2.3%)	32 234 868 (3.3%)
Breastfeeding	36 424 280 (1.7%)	26 620 116 (5.2%)	36 080 948 (1.4%)	26 369 276 (3.2%)	36 081 673 (1.4%)	26 370 002 (2.7%)
Zinc - for treatment of diarrhoea	16 296 788 (0.7%)	4 045 301 (0.8%)	16 378 662 (0.7%)	4 236 493 (0.5%)	23 874 604 (0.9%)	11 732 435 (1.2%)
Rotavirus	16 171 464 (0.7%)	−4 090 655 (−0.8%)	12 644 055 (0.5%)	−7 442 129 (−0.9%)	7 898 913 (0.3%)	−12 187 271 (−1.3%)
Vitamin A supplementation	6 205 260 (0.3%)	484 905 (0.1%)	7 018 875 (0.3%)	1 318 815 (0.2%)	8 272 412 (0.3%)	2 572 352 (0.3%)
Antibiotics - for treatment of dysentery	657 076 (0.03%)	80 262 (0.02%)	760 948 (0.03%)	189 187 (0.02%)	1 079 560 (0.04%)	507 799 (0.1%)
Oral rehydration solution	840 507 (0.04%)	−282 589 (−0.1%)	619 551 (0.02%)	−493 656 (−0.1%)	273 673 (0.01%)	−839 535 (−0.1%)
**Sub total**	136 058 968 (6.2%)	43 417 892 (8.5%)	151 173 443 (6%)	59 317 673 (7.1%)	262 611 005 (9.9%)	170 755 236 (17.7%)
**Total costs**	2 188 346 129 (100%)	508 355 915 (100%)	2 513 796 104 (100%)	834 670 254 (100%)	2 643 406 134 (100%)	964 279 292 (100%)
**Costs per capita**	41	9	47	15	49	18

*Access to safe water includes both an improved water source and a water connection in the home.

## Discussion

This analysis determines the impact and costs of interventions for the prevention and treatment of diarrhoea in children under-five in South Africa. LiST was used to model the impact of scaling up 13 essential interventions between 2014 and 2030. Three scenarios were implemented for linear coverage scale up from baseline (2014): coverage increased 10 percentage points by 2030; coverage increased 20 percentage points by 2030; and interventions reached full coverage (99%) by 2030.

The results show that scaling up diarrhoeal interventions could contribute significantly to the reduction in child mortality in South Africa. In 2030, diarrhoeal deaths are expected to reduce from an estimated 5,500 in 2014 to 2,800 in scenario one, 1,400 in scenario two and 100 in scenario three. The number of diarrhoea cases is also expected to reduce substantially. Approximately five million cases of diarrhoea can be averted by 2030 if interventions are scaled up to full coverage.

This is the first such analysis and there is no recent South African data with which to compare our results. The GHS conducted by Statistics South Africa in 2010 estimated that there were approximately 60 000 cases of diarrhoea per month in children under-five (about 720 000 per year), and 9,000 diarrhoeal deaths (compared to 5,500 deaths in our model) [2]. The District Health Information System (DHIS), which records data at health facility level, estimated that the under-five incidence of diarrhoea was 90.3 per 1000 in 2012 (approximately 520 000 cases) [33]. Though these data sources provide useful information, they may not be entirely representative. The DHIS records the more severe cases of diarrhoea, since a large number of diarrhoea cases are treated at home and/or by traditional healers [34]. Further, it is worth noting that the landscape in the South African health system has changed significantly since the GHS was undertaken. The rotavirus vaccine was introduced in 2009, fewer babies are born HIV positive, and ARV usage has been scaled up [35]. There has also been investment in infrastructure, contributing to the provision of safe water and sanitation to more households. These factors may account for the lower projected diarrhoeal deaths in the model in 2014. While it is difficult to verify the results of our analysis in the absence of updated burden of disease data, the recent under-five mortality estimate indicates that there has been an overall improvement in the burden of childhood morbidity and mortality, and this likely includes diarrhoea [3].

Preventive interventions are crucial. WASH interventions are shown to avert more than 50% of the diarrhoeal deaths, but these also amount to more than 90% of the total intervention costs. Despite many improvements since 1994, South Africa continues to face challenges with implementing home water connections and improved sanitation and there are significant disparities which are not reflected in national statistics. These WASH interventions may thus not be easy to scale up in the timeframe proposed in this analysis. While over 70% of households in South Africa have access to sanitation and over 90% have access to an improved water source, an estimated 12.5% of households in the Eastern Cape province do not have access to any form of sanitation and 14.1% of households in Kwazulu-Natal province have never had access to water [11]. Considerable effort will be required to ensure these services are delivered to the most marginalised, impoverished and at-risk communities. This will require collaboration with the Department of Health and the Department of Water Affairs, as child mortality and in particular diarrhoeal morbidity and mortality cannot solely be resolved through health systems interventions.

This analysis also shows that breastfeeding could save a large number of child lives if full coverage could be achieved. Exclusively breastfed children are 14 times more likely to survive the first six months of life than those who are not breastfed [36], yet South Africa has low exclusive breastfeeding rates [37]. Community peer counselling has been shown to be highly effective in increasing breastfeeding rates in South Africa [38]. However, there are challenges associated with implementing and maintaining such community-based programmes as they require retention of trained health care workers who are adequately remunerated [39], and there are entrenched community practices that are difficult to overcome [40]. Mothers frequently believe that breast milk is insufficient and they give their infants water, gripe water and non-prescription medicines for general health [40]. This increases the risk of developing diarrhoea from contaminated water and may cause children to become undernourished. When diarrhoea does occur, caregivers most commonly choose to treat their infants at home with ORS [34] and many prepare the solution incorrectly [41]. Addressing these behaviour change issues will require considerable effort, community engagement and resources.

The model isolates interventions that impact diarrhoea by maintaining constant coverage of other child health interventions. However, coverage of the other interventions is likely to increase, resulting in a lower burden of under-five mortality by 2030. It is therefore possible that we have overestimated the total number of diarrhoea deaths averted.

This national analysis does not take into account the heterogeneity of intervention coverage and diarrhoeal illness across the 52 districts in South Africa. For example, the institutional diarrhoea case fatality rates in the Eastern Cape are 6.9% compared to the Western Cape at 0.2% [42]. Furthermore, the paper does not address how increased coverage will be achieved, but the estimated intervention costs can guide policy and budget planning. The additional cost of implementing all 13 interventions will range between US$508 million (US$9 per capita) to US$964 million (US$18 per capita) annually. The eight non-WASH interventions would require an additional investment ranging from US$43 million (less than US$1 per capita) to US$170 million (US$3 per capita) per year. These costs are within South Africa’s allocated health budget (approximately US$14.7 billion in 2014/15) [43].

The cost projections are likely an underestimate as staff salary increases were not taken into account and infrastructure development is not fully considered. For the WASH interventions, household infrastructure has been estimated, yet broader system infrastructure requirements, such as waste management systems, have not been considered. Furthermore, the model assumes that the health system interventions are delivered at uniformly high quality. This is unlikely given drug shortages, health care worker attitudes and institutional challenges. Significantly more resources are probably required to address such issues.

HIV/AIDS is a major burden in South Africa. In 2008, approximately 3% of children 0–4 years of age were HIV-positive [44], and this increases the risk of diarrhoeal mortality 11 times [5]. The model does not however explicitly incorporate the relationship between HIV/AIDS and diarrhoea. This should be included in future analyses.

The long term consequences of diarrhoeal illness have not been incorporated in this analysis. Early childhood diarrhoea is associated with impaired physical and cognitive development. The resulting losses in human potential and economic productivity may have a greater impact than the burden of diarrhoeal mortality [45]. Furthermore, the full impact of the 13 interventions, on conditions other than diarrhoea, has not been explored. Increased breastfeeding, for example, is associated with a reduction in several childhood illnesses including respiratory infections, gastroenteritis, otitis media and necrotising enterocolitis [46]. Similarly, other interventions could have benefits in addition to reducing diarrhoea morbidity and mortality. WASH interventions will address neglected tropical diseases which account for significant disability-adjusted life years (DALYs) in low and middle income countries [47], and have significant impacts not only on health, but also on social and economic development [48]. The impact and costs of these additional benefits should be taken into account in future research.

## Conclusion

Diarrhoea is responsible for a substantial number of child deaths in South Africa [2], and reducing its impact could help South Africa attain its post MDG child health targets. This analysis shows that scaling up 13 interventions to full coverage (99%), could prevent more than 5 million diarrhoea cases and 5 000 diarrhoeal deaths in children under-five. Progress has been made by introducing the rotavirus vaccine, adopting the WHO guidelines on infant feeding and attaining the water and sanitation targets of MDG 7. However, there is still a need for increased coverage of exclusive breastfeeding and improved home water and sanitation infrastructure. Given South Africa’s health budget, the cost of scaling up the 13 interventions should be affordable, with the estimated additional costs for the non-WASH health system interventions ranging between under US$1 and US$3 per capita.

## Additional file

Additional file 1:
**Parameters used in the Lives Saved Tool that impact diarrhoea.** Source: Fischer Walker CL, Walker N. The Lives Saved Tool (LiST) as a model for diarrhea mortality reduction. BMC Med. 2014;12:70.
